# OTE-SLAM: An Object Tracking Enhanced Visual SLAM System for Dynamic Environments

**DOI:** 10.3390/s23187921

**Published:** 2023-09-15

**Authors:** Yimeng Chang, Jun Hu, Shiyou Xu

**Affiliations:** School of Electronics and Communication Engineering, Sun Yat-sen University, Shenzhen 518107, China; changym5@mail2.sysu.edu.cn (Y.C.); xushy36@mail.sysu.edu.cn (S.X.)

**Keywords:** visual SLAM, object tracking, dynamic environments

## Abstract

With the rapid development of autonomous driving and robotics applications in recent years, visual Simultaneous Localization and Mapping (SLAM) has become a hot research topic. The majority of visual SLAM systems relies on the assumption of scene rigidity, which may not always hold true in real applications. In dynamic environments, SLAM systems, without accounting for dynamic objects, will easily fail to estimate the camera pose. Some existing methods attempt to address this issue by simply excluding the dynamic features lying in moving objects. But this may lead to a shortage of features for tracking. To tackle this problem, we propose OTE-SLAM, an object tracking enhanced visual SLAM system, which not only tracks the camera motion, but also tracks the movement of dynamic objects. Furthermore, we perform joint optimization of both the camera pose and object 3D position, enabling a mutual benefit between visual SLAM and object tracking. The results of experiences demonstrate that the proposed approach improves the accuracy of the SLAM system in challenging dynamic environments. The improvements include a maximum reduction in both absolute trajectory error and relative trajectory error by 22% and 33%, respectively.

## 1. Introduction

The Simultaneous Localization and Mapping (SLAM) technique is widely used to estimate the pose of a robot and reconstruct the structure of the environment in real-time, without any prior knowledge about the environment. Visual SLAM, in particular, leverages cameras as the source of measurements.

Over the past few decades, extensive research has been conducted and several outstanding visual SLAM algorithms have been designed, such as ORB-SLAM2 [1], a feature-based method, DSO [2], a direct method, and SVO [3], a semi-direct method, PL-SLAM [4], a method using point and line features and CNN-SLAM [5], a method with CNN-based depth prediction. These state-of-the-art visual SLAM systems demonstrate impressive performance in static environments. However, in the real world, environments often do not conform to the assumption of scene rigidity, and the presence of dynamic objects can interfere with the accuracy of conventional SLAM systems. As a result, the estimation accuracy of these systems tends to degrade.

Typically, conventional SLAM algorithms employ outlier removal techniques such as RANSAC [6] (Random Sample Consensus) to mitigate the impact of dynamic objects in the scene. However, these methods are susceptible to failure in scenarios where dynamic objects occupy a substantial portion of the scene. For example, in a busy road, there are many dynamic objects in front of the camera, such as cars, pedestrians, and bicycles. When most of the features are dynamic, the outlier removal techniques may erroneously identify static features as outliers and discard them, leading to a wrong camera pose estimation. Hence, it is imperative to explicitly address the dynamic objects in order to enhance the robustness of the visual SLAM system.

One straightforward approach to address this problem is to detect the presence of dynamic objects and mitigate their impact on SLAM tracking and mapping. Many dynamic SLAM algorithms employ strategies for excluding dynamic features, which involve detecting dynamic regions in the input image and using only features from the static background for camera ego motion tracking [7,8,9,10,11]. However, these approaches may encounter challenges in environments with high dynamics, such as busy roads, where the availability of static features for tracking may be insufficient, leading to potential failures in camera pose tracking.

Limited research has been conducted on exploiting the motion information of dynamic objects in visual SLAM [12,13,14]. These studies can be categorized into two types: loosely coupled methods and tightly coupled methods. Loosely coupled methods optimize the poses of the camera and dynamic objects separately, without leveraging the mutual benefits between SLAM and object tracking. On the other hand, tightly coupled methods integrate the two tasks. Furthermore, the motion information of dynamic objects is valuable for certain applications, such as autonomous driving, where knowledge of the motion status of other vehicles is crucial for making high-level decisions.

In order to address the aforementioned issues, we propose OTE-SLAM, an object tracking enhanced visual SLAM system, which has the advantages of dynamic features removal, while also tracking the motion of dynamic objects. We combine the object tracking and visual SLAM into a tightly coupled system, which jointly optimizes the camera pose, the 3D position of the dynamic objects and the 3D map points in the local bundle adjustment module.

The proposed method is based on the ORB-SLAM2 [1] framework. The YOLOv5-seg network is used to detect the dynamic objects and provide segmentation masks of the objects in each new frame. Consequently, we classify the features extracted from regions containing mobile objects as dynamic features, while those originating from the background and static objects are designated as static features. And, the detected objects are then tracked by the ByteTrack [15] object tracker. We compute the initial position of each object by the camera pose estimated by the tracking module of ORB-SLAM2 and the dynamic features of the object. And, we perform a novel joint optimization of the camera pose, the 3D position of the dynamic objects and the 3D map points in the local bundle adjustment module. The contributions of this paper are summarized as follows:

- The YOLOv5-seg-based dynamic object detection method, which detects the dynamic objects in the image and provides segmentation masks of the objects.
- The ByteTrack-based object tracking method which tracks the dynamic objects and provides data association between segmented instances in consecutive frames.
- An object position estimation method, which computes the initial position of the dynamic objects based on the camera pose and object tracking.
- A novel object-aware and tightly coupled bundle adjustment (BA) framework, which jointly optimizes the poses of the camera and the dynamic objects, and the 3D map points positions.
- A visual SLAM system for dynamic environments, which can operate robustly in dynamic environments with the ability to track the motion of dynamic objects.

The remainder of this paper is structured as follows. Section 2 provides a comprehensive review of related work. In Section 3, we present an overview of the system. Section 4 delves into a detailed exposition of the proposed methodology. Section 5 presents and analyzes the experimental results. Finally, in Section 6, we draw conclusions from our work and explore potential directions for future research.

## 2. Related Work

### 2.1. Dynamic Features Removal

A variety of dynamic features removal methods have been proposed in the literature. These methods typically involve the detection of dynamic features, which are subsequently removed from the feature set used for camera pose tracking. Dynamic objects are treated as outliers that can adversely affect the performance of the SLAM system. The identification of dynamic features predominantly encompasses three classifications: geometric, semantic, and a combination of both. In recent years, artificial intelligence techniques have effectively addressed challenges within the fields of vehicles and robotics [5,12,13,14,16]. Owing to the progressions in deep learning-oriented object detection and semantic segmentation, the procurement of precise and efficient semantic data has been rendered feasible. As a result, recent dynamic SLAM algorithms have increasingly incorporated deep learning methods into their frameworks.

A considerable amount of literature on dynamic object removal has been published. Among these works, DS-SLAM by Yu et al. [7], which builds upon ORB-SLAM2, employs SegNet [17] for semantic segmentation of input images. This method fuses the segmentation results with a moving consistency check approach, based on optical flow, to identify the dynamic features, and utilizes only static features for tracking. Bescos et al. [8] introduced DynaSLAM, which extracts semantic information using Mask R-CNN [18] to detect potentially dynamic objects, i.e., objects that are capable of movement but may not be in motion. A multi-view geometry approach assists in detecting moving objects that are not a priori movable. Moreover, DynaSLAM can inpaint occluded backgrounds with previously acquired static information. Zhong et al. [9] proposed Detect-SLAM, which integrates object detection and SLAM to create a mutually beneficial system. In their paper, the authors present a SLAM-enhanced object detector that leverages geometric information from the reconstructed 3D context to improve performance. Xiao et al. [10] developed Dynamic-SLAM, which incorporates a missed detection compensation algorithm to augment the recall of the object detector. Chang et al. [19] use YOLOv4-tiny network to detect the dynamic regions in the image. In order to tackle the problem of insufficient static features, their method fuses point and surface features to track the camera pose. Due to YOLOv4-tiny’s fast inference speed, their method runs at average 34 FPS with a CPU. Zang et al. [20] proposed an adaptive feature selection method, which is based on the number of dynamic objects and the percentage occupied by them. And, they use Lucas–Kanade optical flow and the RANSAC algorithm to determine the dynamic regions. PLDS-SLAM [21], proposed by Yuan et al., uses line features as additional information, which solves the deficiency of features due to excessive feature removal. The line-segment-based geometric constraint algorithm in their paper improves the camera pose estimation in high dynamic environments.

### 2.2. SLAM with Moving Object Tracking

Recent efforts have been made to leverage object motion information. Yang et al. [22] proposed CubeSLAM, one of the pioneering studies that integrate semantic object detection and geometric SLAM within a unified framework. In their algorithm, objects serve as geometric and scale constraints in bundle adjustment (BA) and help to initialize the depth of points that are challenging to triangulate. Ballester et al. [14] introduced DOT, which tracks objects by minimizing photometric re-projection error to enhance segmentation accuracy. DynaSLAM II, by Bescos et al. [12], tightly integrates multi-object tracking (MOT) and SLAM, jointly optimizing the trajectories of both the camera and moving objects through bundle adjustment. They also proposed an object-centric representation of dynamic points to reduce the number of parameters. Zhang et al. [13] developed VDO-SLAM, which employs instance segmentation and optical flow estimation for a robust estimation, with the ability to handle occlusions caused by the segmentation failure. Fan et al. [11] proposed Blitz-SLAM, which concentrates on eliminating noise blocks generated by moving objects in the point cloud map. Qiu et al. [23] proposed AirDOS, which models the dynamic objects, such as vehicles and pedestrians, as articulated objects, and leverages both the rigidity and the motion of the articulated objects as constraints to improve the robustness of the SLAM system. Liu et al. [24] proposed a switching coupled backend for SLAM and MOT, which does not simply perform loose coupled or tightly coupled method to fuse SLAM and MOT, but switches between them according to the object uncertainty. Sun et al. [25] proposed a Visual SLAM system with multi-objective localization and mapping ability, which uses a depth map and ORB-SLAM2 camera pose to compute the object positions. They use the Deepsort [26] MOT algorithm to associate the masks of the objects.

## 3. System Overview

OTE-SLAM is built on the well-known, fast, and accurate ORB-SLAM2 framework. In this chapter, we will introduce the ORB-SLAM2 framework and an overview of the proposed OTE-SLAM system.

### 3.1. ORB-SLAM2 Framework

ORB-SLAM2 is an open source SLAM system that achieves state-of-the-art performance in static environments, which is developed on the basis of its predecessor ORB-SLAM [27].

It is a feature-based visual SLAM system that uses ORB (Oriented FAST and Rotated BRIEF) features throughout the entire pipeline. Because of its good performance, ease of integration, and open source, it is chosen as the base framework of many dynamic SLAM systems [7,8,10,11,12,22,24,25]. It is also the base framework of our system.

**Figure 1:**
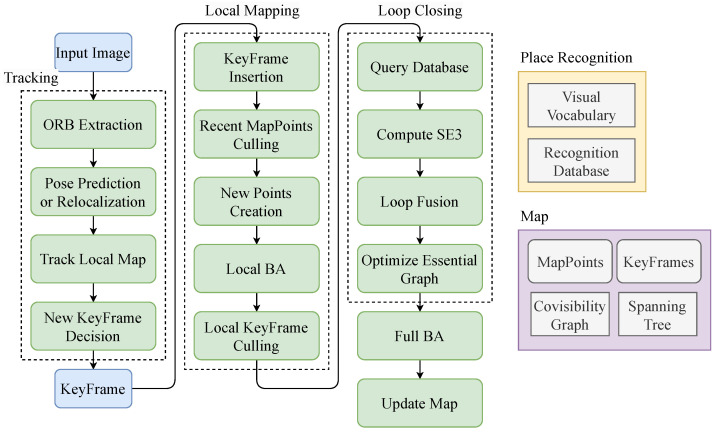
ORB-SLAM2 framework.

The system consists of three threads: tracking, local mapping, and loop closing. In this paper, we use the stereo implementation of ORB-SLAM2. The tracking thread tracks the camera pose and creates new keyframes when necessary. The local mapping thread creates new map points, performs local bundle adjustment, and removes redundant keyframes and map points. The loop closing thread detects loops using bag-of-words (BoW) and performs global bundle adjustment. The framework of ORB-SLAM2 is illustrated in Figure 1.

### 3.2. OTE-SLAM Overview

To improve the performance of ORB-SLAM2 in dynamic environments, we have introduced a new thread for dynamic object detection and tracking. In the tracking thread, only static features are used for camera ego-motion estimation, similar to other dynamic feature removal methods. However, instead of discarding dynamic features, they are retained to represent dynamic objects. The bundle adjustment has been modified to optimize the positions of dynamic objects as well.

**Figure 2:**
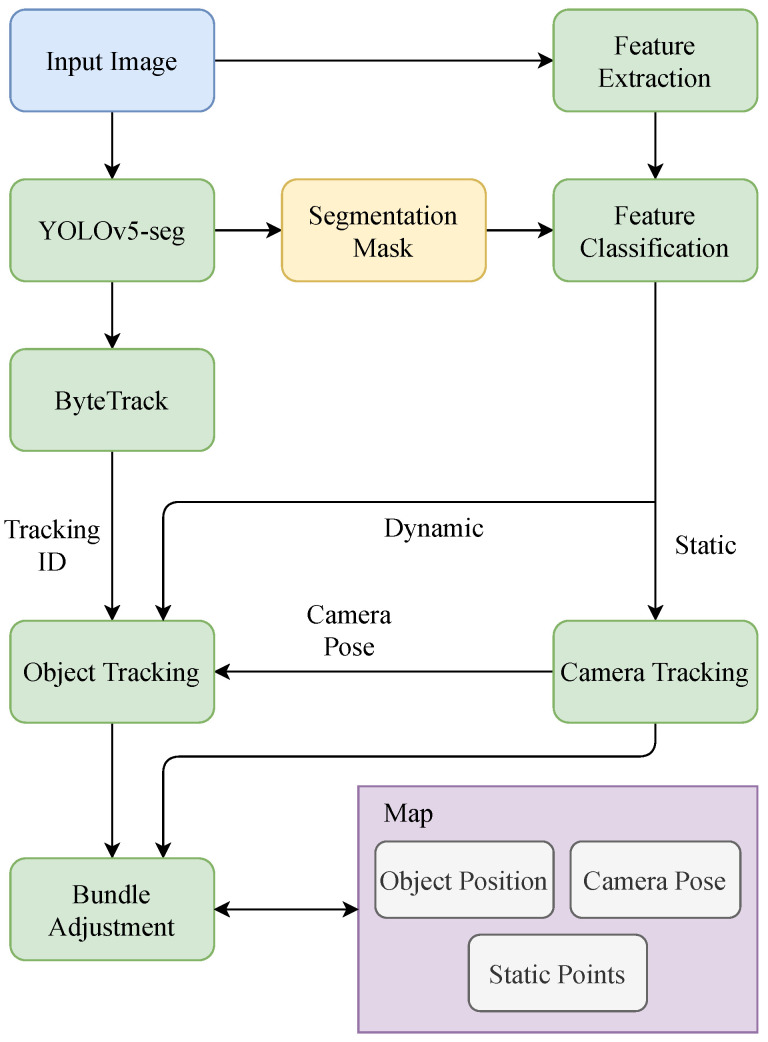
System overview of OTE-SLAM.

The system overview is depicted in Figure 2. First, stereo images are input into the YOLOv5-seg network to obtain bounding boxes and semantic segmentation masks of dynamic objects. In parallel, the feature extraction module extracts ORB features from the images. The segmentation masks are then used to classify features as static or dynamic. Static features are used for camera tracking. The bounding boxes are input into the ByteTrack tracker, which assigns a unique ID to each object. Using the dynamic features and camera pose information, the initial position of each instance in the frame is computed. If a new instance with a new tracking ID is detected, a new object is created in the map, and tracking begins. Finally, the camera pose, 3D object position, and 3D map points are optimized using a bundle adjustment module.

## 4. Methodology

### 4.1. Notations

In this paper, bold lowercase letters are employed to denote vectors, while non-bold lowercase letters are used to represent scalars. Bold capital letters are reserved for denoting matrices. We use Special Euclidean Group in three dimensions (SE(3)) to describe the 3D pose of the camera, which encapsulates all possible rigid body transformations in 3D space, including translation and rotation. The symbols utilized in this paper are summarized as follows:

- ${\left(\cdot \right)}_{W}$, ${\left(\cdot \right)}_{C}$, ${\left(\cdot \right)}_{O}$ represent the world, camera, and object coordinate systems, respectively.
- *i*, *j*, *k* are the indices of the static map points, objects, and frames, respectively.
- $T_{\mathrm{CW}}^{k}\in \mathrm{SE}\left(3\right)$ represents the camera pose at the *k*-th frame, which is a rigid body transformation that transforms points from the world coordinate system to the camera coordinate system.
- $p_{O(\cdot )}^{j,k}\in R^{3}$ represents the position of the *j*-th object at the *k*-th frame in the world coordinate system or camera coordinate system.
- $p_{\left(\cdot \right)}^{i}\in R^{3}$ represents a 3D static point in the world or camera coordinate system.
- $v_{\mathrm{OW}}^{j,k}\in R^{3}$ represents the linear velocity of the *j*-th object at the *k*-th frame in the world coordinate system.

**Figure 3:**
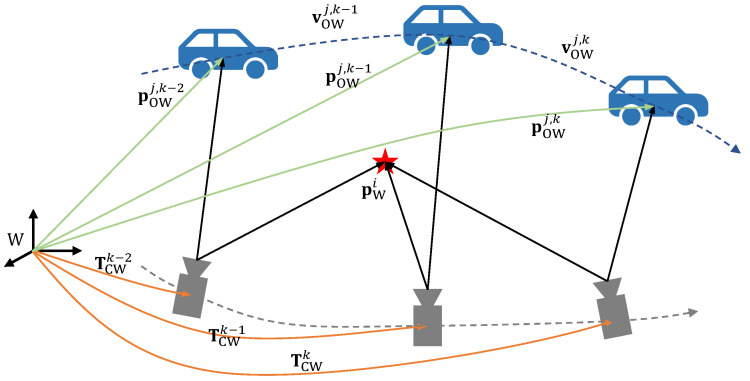
Notations and coordinate systems.

The notations are shown in Figure 3.

In Figure 2, we can see that, compared with ORB-SLAM2, there are some new modules added to the system: a YOLOv5-seg detection and segmentation module, a ByteTrack tracking module, a feature classification module, and an object position estimation module. And, the bundle adjustment module has been modified to optimize the positions of dynamic objects as well. In this chapter, we will introduce these modules in detail.

### 4.2. YOLOv5-Seg Network

**Figure 4:**
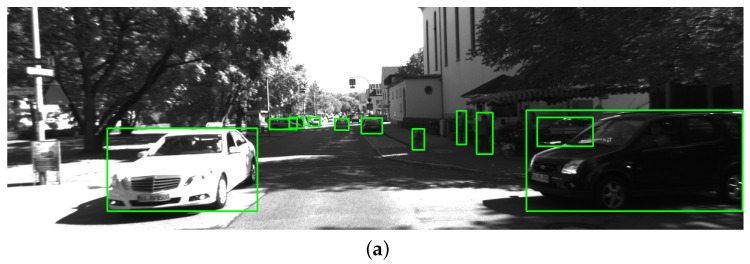

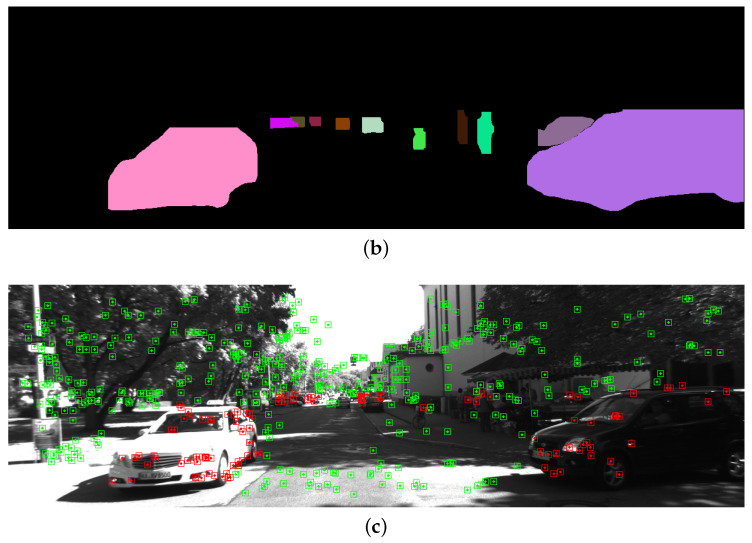
YOLOv5 detection and segmentation result. (**a**) shows the bounding boxes of the detected objects; (**b**) shows the segmentation masks of the objects; (**c**) shows the ORB features extracted from the image. The dynamic objects are marked in red, and the static objects are marked in green.

YOLOv5 is a state-of-the-art object detection network that balances the speed and accuracy of detection, making it suitable for real-time applications. YOLOv5-seg is a modified version of the YOLOv5 architecture. A small fully connected neural network is added to the object detection head to predict segmentation masks of the detected objects. It is a three-layer network that uses the SiLU activation function. Figure 4 shows the detection and segmentation results of the YOLOv5-seg network. We use YOLOv5-seg to process each left image in the stereo pair. It outputs both the bounding box and segmentation mask of each detected object. For object classes with a high probability of being dynamic, such as pedestrians, cars, and trucks, we track them using ByteTrack. For static objects, we treat them as part of the background and do not track them.

### 4.3. ByteTrack Multi Object Tracking

**Figure 5:**
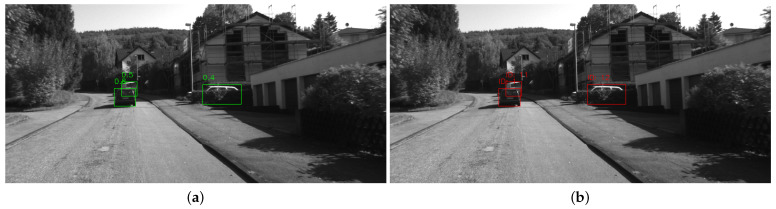
YOLOv5 detection and ByteTrack tracking result. In (**a**), there are three cars detected by YOLOv5, and two of them are occluded by either another car or the plant. ByteTrack can track all of the three cars well, including those with low detection scores, as shown in (**b**).

ByteTrack [15] is a multi-object tracking (MOT) method based on the tracking-by-detection paradigm, which can be easily integrated into our system. It uses the bounding boxes from the YOLOv5 network as input. Most MOT methods only track objects with high detection scores, while ByteTrack tracks by associating almost all detection boxes in the frame, which enables it to handle occluded objects well. For low-score bounding boxes, ByteTrack uses their similarities to recover the true objects. Figure 5 shows the ability of ByteTrack to handle occlusion.

### 4.4. Feature Classification

We classify features as dynamic or static based on the YOLOv5-seg segmentation results. Algorithm 1 outlines the feature classification process. ORB features are first extracted from the left image. Stereo matching is then performed to obtain depth information for all features. ORB features are classified as dynamic or static based on the segmentation mask. Dynamic features are used for computing object position, while static features are used for camera pose estimation.
**Algorithm 1** Feature Classification1:**Input:** Input image, camera intrinsic matrix2:**Output:** Dynamic features of each instance, static features, depth of each feature3:Extract ORB features from the input image4:Stereo matching to obtain the depth of each feature5:YOLOv5-seg segmentation to obtain the segmentation mask of each pixel6:**for** each instance mask **do**7:   **for** each feature **do**8:       **if** the feature is in the segmentation mask **then**9:        Add the feature to the dynamic feature list of the instance10:     **else**11:        Add the feature to the static feature list12:     **end if**13:   **end for**14:**end for**

### 4.5. Object Position Estimation

To obtain the 3D object position, an association between 2D instance masks and 3D objects is necessary. We achieve this by utilizing the ORB features located in the masks. For each new frame, ORB features are extracted and classified as static or dynamic based on the semantic masks of potential dynamic objects. Static features are used for camera pose estimation, while dynamic features are used to compute object position. In the tracking module, when a new instance with a track ID is detected, we first compute its 3D position in the camera coordinate system. For stereo cameras, key point depth has already been computed by stereo matching. We use the 2D pixel coordinates of key points, their depth, and the camera intrinsic matrix to compute the 3D position of the object. The center of mass of all corresponding 3D points of key points in the instance mask is used as the 3D position of the object. First, the 2D key points are projected to the camera coordinate system. For a pixel coordinate $x^{i,k}=\left(u,v\right)$, the 3D position of the key point is computed as:

(1) $$p_{C}^{i,k}=\left[\begin{array}{c} x_{C}^{i,k}\\ y_{C}^{i,k}\\ z_{C}^{i,k} \end{array}\right]=\left[\begin{array}{c} \frac{u-c_{x}}{f_{x}}\\ \frac{v-c_{y}}{f_{y}}\\ 1 \end{array}\right]d^{i,k}$$

where $f_{x}$ and $f_{y}$ are the focal length of the camera, and $c_{x}$ and $c_{y}$ are the principal point of the camera. $d^{i,k}$ is the depth of the key point $x^{i,k}$. Then, the position of the object is computed as the center of mass of all of the key points in the instance mask:

(2) $$p_{\mathrm{OC}}^{j,k}=\frac{1}{N}\sum_{x^{i,k}\in M^{j,k}}p_{C}^{i,k}$$

where $M^{j,k}$ is the set of all the key points in the instance mask of the *j*-th object at the *k*-th frame, and *N* is the number of the key points in the instance mask. The 3D position of the object in the world coordinate system is computed as:

(3) $$p_{\mathrm{OW}}^{j,k}=T_{\mathrm{CW}}^{k}p_{\mathrm{OC}}^{j,k}$$

When an instance with an existing track ID is detected, we compute the linear velocity of the object by its position in the previous frame and the current frame. The linear velocity is computed as:

(4) $$v_{\mathrm{OW}}^{j,k}=\frac{p_{\mathrm{OW}}^{j,k}-p_{\mathrm{OW}}^{j,k-1}}{\Delta t}$$

where Δt is the time interval between the two frames. When an object in the map is not detected in a new frame, we do not remove it immediately. Instead, we keep it in the map and predict its position in the new frame by assuming a constant linear velocity. If the object is missing for more than a certain number of frames, it will be removed from the map. The object position initialization and tracking strategy is shown in Algorithm 2.
**Algorithm 2** Object position initialization and tracking strategy1:**Input:** Instance masks, track IDs, camera pose, ORB features of each instance2:**Output:** Object positions, linear velocities3:Update the ByteTrack tracking results4:**for** each detected instance in the new frame **do**5:     **if** the track ID of the instance is not in the map **then**6:       **if** the instance has enough features **then**7:          Compute the position of the object in the camera coordinate system8:          Compute the position of the object in the world coordinate system9:          Initialize the linear velocity of the object as zero10:        Add the object to the map11:     **else**12:        Compute the position of the object in the camera coordinate system13:        Compute the position of the object in the world coordinate system14:        Compute the linear velocity of the object15:     **end if**16:   **end if**17:**end for**18:**for** each object in the map **do**19:   **if** the position of the object is not updated in the new frame **then**20:     **if** the object is missing for more than a certain number of frames **then**21:        Remove the object from the map22:     **else**23:        Predict the position of the object in the new frame24:     **end if**25:   **end if**26:**end for**

### 4.6. Joint Optimization

**Figure 6:**
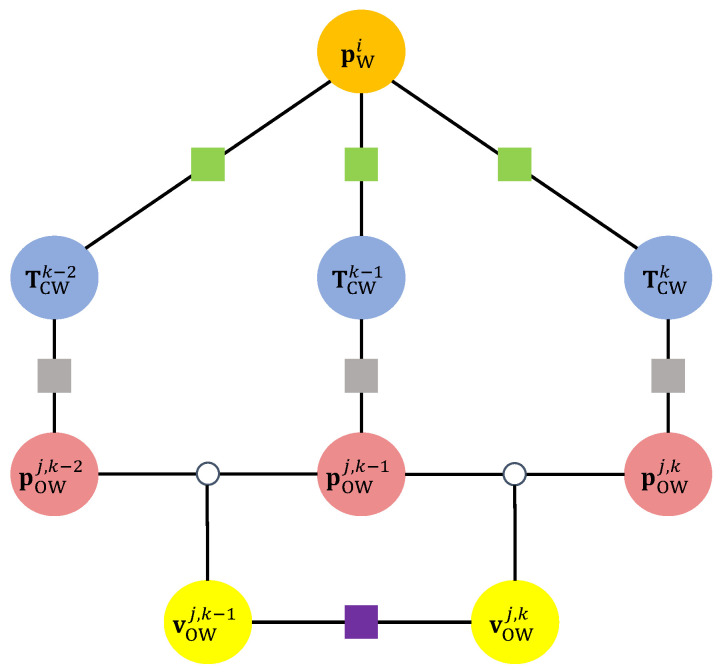
Factor graph of the optimization problem. The green squares represent the re-projection error terms for the static points. The gray squares represent the re-projection error terms for the dynamic objects. The purple squares represent the object motion smoothness regularization terms.

ORB-SLAM2 performs local bundle adjustment to optimize the camera pose and static map points. In our method, we extend local bundle adjustment to optimize the camera pose, static map points, and dynamic object positions. The factor graph of the bundle adjustment problem is shown in Figure 6. We minimize the re-projection error of static points to optimize the camera pose and static map points. We also define a re-projection error term for dynamic objects to optimize the camera pose and dynamic object positions. Additionally, we define a motion smoothness regularization term to regularize the motion of dynamic objects.

#### 4.6.1. Static Points Re-Projection Error

The re-projection error term for the static points is defined the same way as in ORB-SLAM2, as shown in Equation (5). It represents the difference between the 2D pixel coordinates of static points and the projection of 3D static points in the camera coordinate system onto the image plane. The only difference is that only static points are used in this error term. This error term is used to optimize the camera pose and static map points.

(5) $$e^{i,k}=x^{i,k}-\pi_{k}\left(T_{\mathrm{CW}}^{k}P_{W}^{i}\right)$$

where $x^{i,k}\in R^{2}$ is the 2D image coordinate of the map point *i* in the keyframe *k*, $T_{\mathrm{CW}}^{k}\in SE\left(3\right)$ is the camera pose of the keyframe *k* in the world coordinate, $P_{W}^{i}\in R^{3}$ is the 3D position of the map point *i* in the world coordinate, and $\pi_{k}$ is the projection function of the camera *k*:

(6) $$\begin{array}{c} \pi_{k}\left(T_{\mathrm{CW}}^{k}P_{W}^{i}\right)=\left[\begin{array}{c} f_{x}\frac{x^{i,k}}{z^{i,k}}+c_{x}\\ f_{y}\frac{y^{i,k}}{z^{i,k}}+c_{y} \end{array}\right]\\ \left[\begin{array}{c} x^{i,k}\\ y^{i,k}\\ z^{i,k} \end{array}\right]=T_{\mathrm{CW}}^{k}P_{W}^{i} \end{array}$$

The bundle adjustment minimizes the re-projection error of all the map points in the keyframe and its neighbors in the co-visibility graph. The optimization problem is formulated as:

(7) $$\left\{T_{\mathrm{CW}}^{k},P_{W}^{i}\right\}=\arg\min_{T_{\mathrm{CW}}^{k},P_{W}^{i}}\sum_{i,k}\rho_{\mathrm{Huber}}\left({e^{i,k}}^{T}{\left(\Omega_{s}^{i,k}\right)}^{-1}e^{i,k}\right)$$

where $\Omega_{s}^{i,k}$ is the covariance matrix of the re-projection error, and $\rho_{\mathrm{Huber}}\left(\cdot \right)$ is the Huber loss function, which is defined as follows:

(8) $$\rho_{\mathrm{Huber}}\left(a\right)=\left\{\begin{array}{cc} \frac{1}{2}a^{2} & \;\mathrm{for}\;|a|\leq \delta \\ \delta \cdot \left(\left|a\right|-\frac{1}{2}\delta \right) & \;\mathrm{otherwise}.\; \end{array}\right.$$

The Huber loss function serves as a compromise between mean squared error (MSE) and mean absolute error (MAE). It behaves quadratically (like MSE) for small absolute differences (within a threshold δ) to capture fine-grained differences. When absolute differences exceed δ, it transitions to a linear behavior (like MAE) for robustness against outliers, penalizing them with a constant penalty determined by δ. This property makes it suitable for handling noisy data with outliers.

Here, we provide a detailed explanation of the covariance matrix $\Omega_{s}^{i,k}$. For a specific observation $z_{i,k}$, we have:

(9) $$z_{i,k}=\pi_{k}\left(T_{\mathrm{CW}}^{k}P_{W}^{i}\right)+v_{i,k}$$

where $v_{i,k}$ is the noise item. We assume that this noise item follows a Gaussian distribution, denoted as $v_{i,k}∼N\left(0,\Omega_{s}^{i,k}\right)$. So, the conditional probability of the observation is:

(10) $$P\left(z_{i,k}|T_{\mathrm{CW}}^{k},P_{W}^{i}\right)=N\left(\pi_{k}\left(T_{\mathrm{CW}}^{k}P_{W}^{i}\right),\Omega_{s}^{i,k}\right)$$

which is still a Gaussian distribution. It implies that the maximum likelihood estimation of $T_{\mathrm{CW}}^{k},P_{W}^{i}$ can be acquired using Equation (7). Equation (7) is equivalent to a quadratic form that minimizes the Mahalanobis distance. It can also be regarded as the Euclidean distance weighted by ${\left(\Omega_{s}^{i,k}\right)}^{-1}$, which is called the information matrix.

#### 4.6.2. Dynamic Objects Re-Projection Error

The re-projection error term for dynamic objects also involves a 2D–3D association. However, it operates on a higher level than the ORB features and map points. Instead of associating ORB features and map points, we define the error term as the difference between the 2D instance mask position and the projection of the 3D object position in the camera coordinate system onto the image plane:

(11) $$e_{\mathrm{object}}^{j,k}=x^{j,k}-\pi_{k}\left(T_{\mathrm{CW}}^{k}p_{\mathrm{OW}}^{j,k}\right)$$

where $x^{j,k}$ is the 2D pixel coordinates of the instance corresponding to the *j*-th object at the *k*-th frame, which is defined as the center of mass of the ORB features in the instance mask.

#### 4.6.3. Object Motion Smoothness Regularization

We assume that the object moves in a constant linear velocity. To make the estimated object motion smooth, we add a regularization term to the optimization problem:

(12) $$e_{\mathrm{smooth}}^{j,k}=m_{O}^{j,k}-m_{O}^{j,k-1}$$

Let $\theta =\{T_{\mathrm{CW}}^{k},p_{\mathrm{OW}}^{j,k},p_{W}^{i},m_{O}^{j,k}\}$ be the optimization variables. The optimization problem is defined as

(13) $$\begin{array}{cc} \theta & =\arg\min_{\theta }\sum_{i,k}\rho_{h}\left({e_{\mathrm{static}}^{i,k}}^{T}{\left(\Omega_{s}^{i,k}\right)}^{-1}e_{\mathrm{static}}^{i,k}\right)\\ \; & +\sum_{j,k}\rho_{h}\left({e_{\mathrm{dynamic}}^{j,k}}^{T}{\left(\Omega_{o}^{j,k}\right)}^{-1}e_{\mathrm{dynamic}}^{j,k}\right)\\ \; & +\sum_{j,k}\rho_{h}\left({e_{\mathrm{smooth}}^{j,k}}^{T}{\left(\Omega_{m}^{j,k}\right)}^{-1}e_{\mathrm{smooth}}^{j,k}\right) \end{array}$$

where $\Omega_{s}^{i,k}$, $\Omega_{o}^{j,k}$ are the covariance matrices of the static points and the dynamic objects, respectively. $\Omega_{m}^{j,k}$ is the covariance matrix of the object motion smoothness.

In alignment with the approach employed in the original ORB-SLAM2 framework, we define $\Omega_{s}^{i,k}=\sigma_{i,k}^{2}I_{2\times 2}$, where $\sigma_{i,k}$ is the level within the image pyramid from which this feature is extracted. This choice is made based on the observation that noise tends to decrease for finer layers of the pyramid. And, for the features from finer levels, $1/\sigma_{i,k}^{2}$ is bigger so that this error item is assigned greater weight in the optimization. This adjustment is motivated by the higher reliability of features obtained from finer levels, making them more influential in the estimation process compared to features from coarser levels. As for $\Omega_{o}^{j,k}$ and $\Omega_{m}^{j,k}$, we opt to set them as identity matrices. This choice is motivated by the absence of enough prior information regarding the noise characteristics for these components.

## 5. Experiments

The experiments are conducted on a laptop with an Intel Core i9-10855H CPU and an NVIDIA Quadro T2000 GPU. The system is Ubuntu 18.04. We run the YOLOv5-seg network with CUDA 11.7 for GPU acceleration. The pre-trained weights from the YOLOv5-seg repository is used, which is trained on the COCO dataset.

We evaluate the performance of our SLAM system using the KITTI dataset [28], which is a widely used dataset for autonomous driving research. It contains recorded road scenes with moving cars, pedestrians, cyclists, and other dynamic objects. The dataset includes camera images, laser scans, GPS measurements, and IMU accelerations. Ground truth poses of the camera are also provided.

We use two metrics to evaluate performance. The first is the absolute pose error (APE), which assesses the absolute accuracy of pose estimates by comparing them to ground truth or reference poses. It quantifies the overall spatial error between estimated poses and their corresponding ground truth. It is notable that we use the Kabsch–Umeyama algorithm to align the two trajectories before computing the APE. The absolute relative pose in frame *k* between the ground truth pose $T_{\mathrm{gt}}^{k}\in \mathrm{SE}\left(3\right)$ and estimated pose $T_{\mathrm{est}}^{k}\in \mathrm{SE}\left(3\right)$ is defined as:

(14) $$E_{k}={\left(T_{\mathrm{gt}}^{k}\right)}^{-1}T_{\mathrm{est}}^{k}\in \mathrm{SE}\left(3\right)$$

In this paper, we compare the APE translation error. The APE of the translation part of the estimated pose is computed as following:

(15) $${APE}_{\mathrm{trans}}^{k}=∥trans(E_{k})∥$$

where trans(·) computes the translation part of the transformation matrix.

The second metric is the relative pose error (RPE), which is used to quantify the relative pose error between consecutive frames, typically employed for assessing the accuracy of trajectory estimation. It measures the discrepancy in pose estimates between sequential frames. The delta pose difference is defined as:

(16) $$E_{k,\Delta }={\left({\left(T_{\mathrm{gt}}^{k}\right)}^{-1}T_{\mathrm{gt}}^{k+\Delta }\right)}^{-1}\left({\left(T_{\mathrm{est}}^{k}\right)}^{-1}T_{\mathrm{est}}^{k+\Delta }\right)\in \mathrm{SE}\left(3\right)$$

The RPE is measured in percent (for translation) and in degrees per 100 m (for rotation). The RPE for the rotation part is computed as follows:

(17) $${RPE}_{\mathrm{rot}}^{k,\Delta }={∥rot(E_{k,\Delta }-I_{3\times 3})∥}_{F}$$

The proposed method is compared with the original ORB-SLAM2 algorithm’s stereo implementation and ORB-SLAM2 with dynamic feature removal to demonstrate the improvement. We also compare it with DynaSLAM, a state-of-the-art dynamic SLAM algorithm.

**Figure 7:**
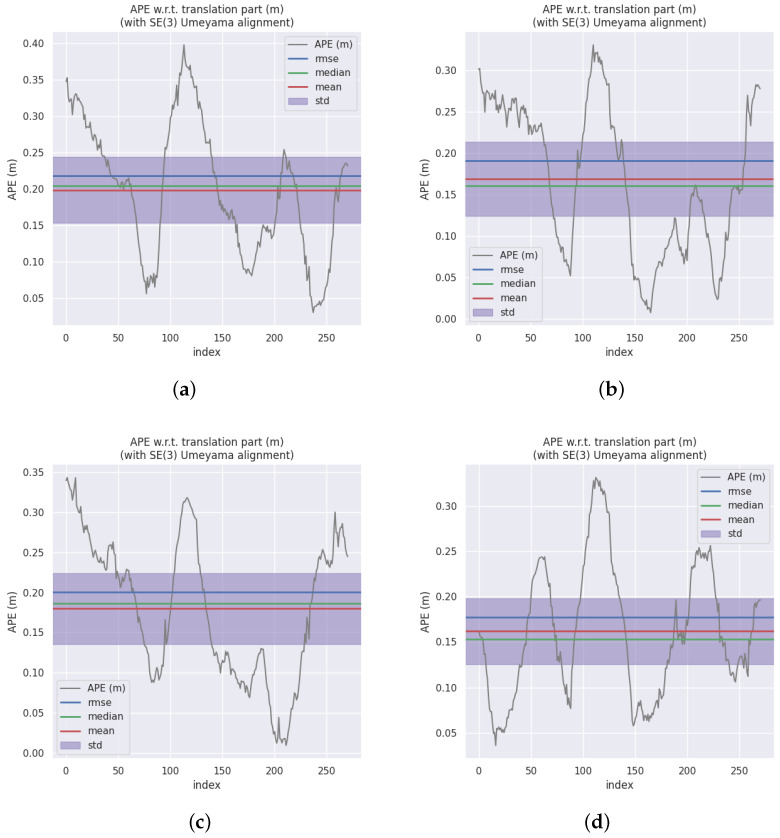
The APE comparison of ORB-SLAM2 and OTE-SLAM on Sequence 04. (**a**) ORB-SLAM2. (**b**) ORB-SLAM2 with masks. (**c**) DynaSLAM. (**d**) OTE-SLAM.

Table 1 shows the root mean square error (RMSE), mean, and standard deviation (STD) of APE for the methods. The RMSE and mean represent the overall performance of the method, while STD shows the robustness of the method. The best results for each sequence are shown in bold. Results demonstrate that OTE-SLAM improves the performance of ORB-SLAM2 on most sequences. This improvement is significant on Sequence 04, in which a car drives in front of the camera for an extended period. ORB-SLAM2 tracks with ORB features extracted from the car region, leading to some incorrect geometric information. By simply removing features in segmentation masks, ORB-SLAM2 improves performance by removing dynamic key points. Meanwhile, OTE-SLAM tracks dynamic objects, making better use of object motion information. As shown in Figure 7, comparison of the APE shows that OTE-SLAM has lower error.

Based on the empirical findings, it can be deduced that ORB-SLAM2 exhibits superior performance in scenarios characterized by minimal scene dynamics, particularly when there are numerous movable objects that are presently stationary (e.g., Sequence 10). Conversely, when there are moving objects whose motion information can be harnessed by OTE-SLAM, OTE-SLAM outperforms ORB-SLAM2, as exemplified in Sequence 04. However, in cases where certain objects exhibit suboptimal feature quality, potentially stemming from erroneous depth estimation or inaccurate segmentation, this may lead to imprecise object pose estimations. In such situations, ORB-SLAM2 (masked) may outperform OTE-SLAM, as observed in the beginning of Sequence 08. This underscores the critical importance of accurate depth estimation and semantic segmentation for object pose estimation.

Table 2 shows the RPE(%) and RPE(deg/100) of the methods on the KITTI dataset. Results demonstrate that the four methods have similar performance on most sequences. In some high dynamic sequences where most detected objects are moving, such as Sequence 04, OTE-SLAM has better performance than the original ORB-SLAM2. But, for some sequences with a relatively small number of dynamic objects, ORB-SLAM2 performs better. This is because when OTE-SLAM tracks the camera pose, it does not use the features on the potential dynamic objects, though it may be static at the moment. In this case, the additional constraints from the dynamic objects are not enough to compensate for the loss of information from the static features. In the future, we will explore how to better utilize the information of movable but static objects to improve the performance of OTE-SLAM.

**Figure 8:**
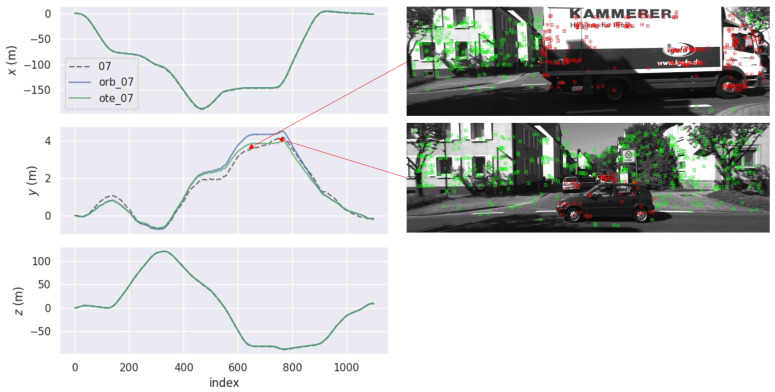
The APE comparison of ORB-SLAM2 and OTE-SLAM on Sequence 07 in the x, y, and z directions. In the two frames shown in the Figure, some vehicles pass by. The green and red points are the static and dynamic features, respectively.

Figure 8 shows the APE comparison of ORB-SLAM2 and OTE-SLAM on Sequence 07 in the x, y, and z directions. From the 500th frame to the 800th frame, OTE-SLAM has performance which is obviously better than ORB-SLAM2 in the y direction. Two frames in this period are shown in the Figure. We can see that the car holding the camera is waiting at the crossroad. While it is waiting, some trucks and cars are passing by. These moving vehicles have a significant impact on the estimation of the camera pose for ORB-SLAM2. Meanwhile, OTE-SLAM does not suffer from this problem because it tracks the camera only with the features in the background and has the object moving information as additional constraints.

**Figure 9:**
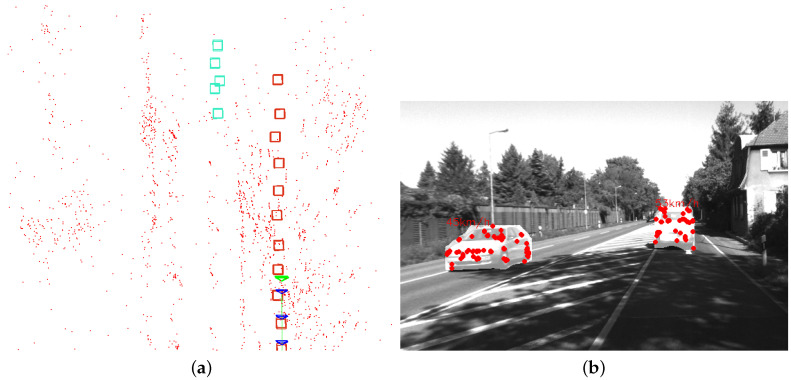
The map with objects created by OTE-SLAM and the corresponding scene in Sequence 04. (**a**) The map with a car driving in front of the camera, and another driving in the opposite direction. The boxes with different colors represent different objects in the map. The blue triangles represent the camera pose in previous frames, and the green triangle represents the camera pose in the current frame. The red dots represents the map points recovered by the system. (**b**) The corresponding scene with the velocity of the cars.

Figure 9 shows a scene in Sequence 04 where a car is in front of the camera and the map created by OTE-SLAM. The map includes tracked dynamic objects, providing additional information for better estimation by the SLAM system. Additionally, the map with dynamic object positions is useful for some applications requiring both high-level and geometric information.

## 6. Conclusions

In this scholarly exposition, we introduce an adept visual SLAM system tailored to dynamic environments. Leveraging the YOLOv5-seg network, our system adeptly identifies and semantically segments dynamic objects. The ByteTrack algorithm enables object tracking across successive frames. The system computes object positions through a fusion of camera pose data and scene-derived depth features. These object positions undergo optimization via a novel bundle adjustment framework that jointly refines camera poses and map point positions.

Our evaluation of this SLAM system is conducted on the KITTI dataset. Our empirical findings demonstrate its robustness in handling dynamic scenarios, resulting in significantly enhanced localization accuracy when compared with the original ORB-SLAM2. The resultant map, enriched with object positions, caters to applications necessitating both high-level semantic understanding and geometric information.

Nevertheless, it is imperative to acknowledge the existence of certain limitations within our proposed system. Firstly, it may misconstrue movable objects as dynamic, though they remain motionless. Such instances necessitate caution, as our aim is to maximize the inclusion of static features to enhance camera pose estimation. Secondly, the robustness of object position initialization is compromised due to occasional inaccuracies in masking. In our future endeavors, we endeavor to rectify these limitations, thereby rendering our visual SLAM system adaptable to even more challenging environments.

## Figures and Tables

**Table 1:** Comparison of the APE (m).

Sequence	ORB-SLAM2			ORB-SLAM2 (Masked)			DynaSLAM			OTE-SLAM		
	RMSE	Mean	Std	RMSE	Mean	Std	RMSE	Mean	Std	RMSE	Mean	Std
Sequence 01	10.78	10.23	3.39	**9.85**	**9.34**	**3.13**	11.79	10.68	4.98	10.44	9.80	3.61
Sequence 03	0.73	0.61	0.39	0.79	0.67	0.41	0.75	0.64	0.39	**0.62**	**0.52**	**0.34**
Sequence 04	0.22	0.20	0.09	0.19	0.19	0.09	0.20	0.18	0.09	**0.17**	**0.16**	**0.07**
Sequence 06	0.84	0.80	0.26	**0.82**	**0.77**	0.31	0.84	0.82	**0.18**	1.04	1.01	0.24
Sequence 07	0.57	0.54	0.21	0.55	0.52	**0.18**	0.54	0.50	0.20	**0.52**	**0.48**	0.20
Sequence 08	3.46	3.14	1.45	3.87	3.59	1.37	**3.41**	**3.13**	1.35	4.01	3.78	**1.32**
Sequence 10	**1.15**	**1.04**	0.50	1.25	1.15	**0.51**	1.24	1.11	0.55	1.22	1.08	0.54

**Table 2:** Comparison of the RPE (%) and RPE (deg/100 m).

Sequence	ORB-SLAM2		ORB-SLAM2 (Masked)		DynaSLAM		OTE-SLAM	
	RPE	RPE	RPE	RPE	RPE	RPE	RPE	RPE
	[%]	[deg/100 m]	[%]	[deg/100 m]	[%]	[deg/100 m]	[%]	[deg/100 m]
Sequence 01	1.43	0.22	**1.40**	0.19	1.87	**0.17**	1.45	0.20
Sequence 03	0.69	0.20	0.72	**0.16**	0.73	**0.16**	**0.65**	0.20
Sequence 04	0.50	0.12	0.47	0.18	**0.43**	0.11	**0.43**	**0.08**
Sequence 06	**0.54**	0.22	0.56	**0.18**	**0.54**	0.19	0.64	0.21
Sequence 07	0.55	0.29	**0.50**	**0.27**	**0.50**	0.31	0.53	0.30
Sequence 08	1.07	0.33	1.07	**0.32**	1.08	0.33	1.07	**0.32**
Sequence 10	0.64	**0.29**	**0.59**	0.31	0.68	0.35	0.67	0.30

## Data Availability

KITTI dataset used in this paper is available at http://www.cvlibs.net/datasets/kitti (accessed on 26 June 2023).

## Abbreviations

The following abbreviations are used in this manuscript:

SLAM	Simultaneous localization and mapping
MOT	Multi-object tracking
RANSAC	Random sample consensus
ORB	Oriented FAST and Rotated BRIEF
YOLO	You Only Look Once
MSE	Mean squared error
MAE	Mean absolute error
APE	Absolute pose error
RPE	Relative pose error
RMSE	Root mean square error
STD	Standard deviation
